# Preventive Effect and Safety of a Follicle Stimulating Hormone Inhibitory Formulation Containing a Mixture of* Coicis Semen* and* Artemisia capillaris* for Precocious Puberty: A Preliminary Experimental Study Using Female Rats

**DOI:** 10.1155/2017/2906014

**Published:** 2017-11-20

**Authors:** Tuy An Trinh, Seung Chan Park, Jihong Oh, Chang-Eop Kim, Ki Sung Kang, Hwa Seung Yoo, Hye Lim Lee

**Affiliations:** ^1^College of Korean Medicine, Gachon University, Seongnam, Republic of Korea; ^2^Highki Korea Medicine Clinic, 6 Dong 5F, 205, Shinbanpo-ro, Seocho-gu, Seoul, Republic of Korea; ^3^East West Cancer Center, Dunsan Korean Medicine Hospital of Daejeon University, Daejeon, Republic of Korea

## Abstract

**Background:**

Precocious puberty is a common endocrine disease in children. Inappropriate activation of hypothalamic–pituitary–gonadal axis leads to the development of secondary sexual characteristics at an earlier age than normal children and causes short stature in adulthood.

**Objectives:**

The aim of this study is to evaluate the preventive effects of a herbal formulation containing a mixture of* Coicis Semen* and* Artemisia capillaris* (hEIF extract) on precocious puberty.

**Methods:**

The preventive effect of hEIF extract on precocious puberty in rats was evaluated by measuring blood component after 3 weeks of treatment via oral administration. Network pharmacological analyses were performed to predict the bioactive components of hEIF extract.

**Results:**

* In vivo* studies showed that hEIF extract significantly reduced follicle stimulating hormone (FSH) levels. After treatment with 200 mg/kg of hEIF extract, the FSH level was 5.33 ± 1.10 ng/mL, whereas the FSH level in the vehicle group was 46.73 ± 0.80 ng/mL. Moreover, the use of hEIF extract did not stimulate body growth and bone accretion in rats. The network pharmacological analysis led to the identification of multiple targets of hEIF extract related to lipolysis and the female sex hormone-related pathways.

**Conclusion:**

hEIF extract can be used as an FSH inhibitor for the treatment of precocious puberty.

## 1. Introduction

Precocious puberty is defined as the onset of pubertal changes before age 8 in girls and before age 9 in boys. Signs or symptoms of precocious puberty include early pubertal development, rapid bone maturation, decreased adult height, inappropriate body shape, and mental behavioral abnormalities. As the number of patients with precocious puberty increases year after year, more parents have expressed concerns about the negative effect on their children's growth [1]. The early maturation of the hypothalamus–pituitary–gonad (HPG) axis is associated with gonadotropin-dependent (central/true) precocious puberty, and inactivation of the HPG axis is associated with gonadotropin-independent (peripheral/pseudo) precocious puberty [2].

Precocious puberty is 5–10 times more common in girls than in boys. Among girls, approximately 90% of cases are idiopathic, whereas up to 75% of cases among boys are found to involve central nervous system abnormalities. The underlying causes of idiopathic precocious puberty have not been elucidated; the normal puberty mechanism is expressed too early [3]. The majority of precocious puberty cases is idiopathic, but environmental factors that mimic hormones and substrates (endocrine-disrupting chemicals), as well as organic lesions, can exert estrogenic activity or increase endogenous estrogen secretion. Androgen- and estrogen-containing substances, such as estrogen-containing cosmetics and food items, and some pharmacological insecticides can interfere with the HPG axis and result in iatrogenic precocious puberty [4, 5].

Although there is no normal index of puberty in Korea, similar to other countries, the age of menarche and breast development have shown a gradual decrease worldwide. Obesity has been shown to accelerate the age of menarche in girls. The number of high-risk obese children has increased owing to a general reduction in physical activity and an increase in the intake of high-calorie foods, such as fast food [6]. Precocious puberty is usually managed with 4-week treatment with depot formulations that deliver gonadotropin-releasing hormone (GnRH). However, the side effects may include headache, nausea, and facial flushing during treatment [7].

In our ongoing search to find herbal formulations for the treatment of precocious puberty, we identified a possible treatment that comprises a mixture of two herbs,* Semen Coicis* and* Artemisia capillaris* Thunberg* (A. capillaris)*.* Coicis Semen* is a ripe seed of* Coix lacryma-jobi *L. var.* mayuen* Stapf. and has been used for the treatment of diuresis, analgesia, gynecological issues, edema, neuralgia, rheumatism, and bladder stones and for its tonic effects [8, 9].* Coicis Semen* is reported to contain stigmasterol, sitosterol, vitamin B1, vitamin E, linoleic acid, and other active ingredients that are effective against obesity [8].* A. capillaris*, a perennial mugwort from the Asteraceae family [10], is reported to promote liver function and fat decomposition [11]. Quercetin, eupalitin, sitosterol, vitamin A, and various minerals are found in* A. capillaris* [12, 13]. In this study, we demonstrated the ameliorative potential of* Coicis Semen *and* A. capillaris* formulation administered to female rats with precocious puberty. Moreover, we investigated the mechanism of action of the formulation at systems level by applying network pharmacological analysis. This novel approach offers an opportunity to understand the complex mechanism of the herbal formulations containing multiple compounds.

## 2. Materials and Methods

### 2.1. Chemicals and Reagents

ELISA kits to detect alkaline phosphatase (ALP) activity and the concentrations of estradiol, osteocalcin, IGF-1, IGFBP-3, luteinizing hormone (LH), and follicle stimulating hormone (FSH) were purchased from R&D Systems (Minneapolis, USA).

### 2.2. Preparation of the Herbal Formulation to Prevent Precocious Puberty

Dried* Coicis Semen* and* A. capillaris* were purchased from Kyoungdong Herbal Market, Seoul, in July 2015. The ethanolic extracts of* Coicis Semen* and* A. capillaris* mixture in the ratio 1 : 1 were prepared (herbal estrogen inhibition formulae (hEIF) extract) as a dried powder by Hanpoong Pharmaceutical Co., Ltd. (Seoul, Korea) and used.

### 2.3. Experimental Animal and Experimental Design

In this study, we followed the Guidelines for Animal Experimentation approved by Gachon University. Sprague–Dawley rats with a mean body weight of 110 g were given hEIF extract for 3 weeks, and the effects on precocious puberty were identified. The rats were divided into four groups on the basis of their body weight, and the groups received either the control treatment or hEIF extract at different concentrations. The prepared hEIF extract was administered orally at 50, 100, and 200 mg/kg BW to rats. 

*Group 1*. Normal (*n* = 5), received water only. 

*Group 2*. hEIF 50 (*n* = 5), orally administered hEIF extract (50 mg/kg) as an aqueous solution. 

*Group 3*. hEIF 100 (*n* = 5), orally administered hEIF extract (100 mg/kg) as an aqueous solution. 

*Group 4*. hEIF 200 (*n* = 5), orally administered hEIF extract (200 mg/kg) as an aqueous solution.

### 2.4. Measurement of Body Weight, Food Intake, and Body Length

During the experimental period, the dietary intake and the body weight gain of the rats were measured twice per week. The rat body length was measured horizontally from the nose tip and excluded part of the tail length; however, the total length measurement included the tail as well. To measure the femur length at the end of the experiment, the muscles were removed and the linear length from the femoral head to the distal tip of the femur was measured using a caliper.

### 2.5. Measurement Organ Weight

At the end of the experiment, the dissected organs (liver, kidney, spleen, and ovary) were washed with physiological saline. Moisture was removed with a gauze, and the organ weights were measured.

### 2.6. Blood Component Analysis

Blood samples were collected in test tubes containing 0.18 M EDTA and centrifuged at 5,000 rpm for 5 min at 4°C. The serum ALP activity and concentrations of estradiol, osteocalcin, insulin-like growth factor 1 (IGF-1), IGF binding protein 3 (IGFBP-3), luteinizing hormone (LH), and follicle stimulating hormone (FSH) were analyzed by using the appropriate enzymatic colorimetric kits in accordance with the manufacturer's instructions. Bone mineral density was analyzed using a bone mineral density meter from Doo Yeol Biotech (Seoul, Korea).

### 2.7. Network Pharmacological Analysis

We obtained herbal compound–target gene information from the Traditional Chinese Medicine Systems Pharmacology Database (TCMSP, http://ibts.hkbu.edu.hk/LSP/tcmsp.php) [14] and set oral bioavailability (OB) ≥ 30 [15] and the drug-likeness (DL) index ≥ 0.18 [16] as the minimum thresholds, which were the default values suggested in TCMSP.

We further analyzed pathways of target genes using the Kyoto Encyclopedia of Genes and Genomes database (KEGG, http://www.genome.jp/kegg/) [17]. After collecting information about compounds, targets, and pathways, we integrated the information as a network graph using Cytoscape.

### 2.8. Statistical Processing

The experimental results were expressed as average ± standard error. Statistical analyses were performed using Student's *t*-test or one-way ANOVA. *p* < 0.05 was considered statistically significant.

## 3. Results and Discussion

Precocious puberty is a common endocrine disorder that results in the development of secondary sexual characteristics at an earlier age than normal, before 8 years of age in girls and 9 years of age in boys. The incidence of precocious puberty is higher in girls than in boys [18]. Precocious puberty is classified into central precocious puberty (gonadotropin-dependent) and peripheral precocious puberty (gonadotropin-independent) [19]. The incidence of central precocious puberty in Korean girls has increased significantly from 3.3 per 100,000 girls in 2004 to 50.4 per 100,000 girls in 2010 [20]. The growth spurt that results from precocious puberty terminates quickly, which leads to short stature in adulthood. The early appearance of thelarche or menarche can cause emotional distress in some children.

In gonadotropin-dependent cases, the onset of precocious puberty is initiated by early GnRH secretion from the hypothalamus, which inappropriately activates the hypothalamic–pituitary–gonadal axis. GnRH is released from the hypothalamus by the stimulation of neurokinin B and kisspeptin [18]. GnRH activates the pituitary gland to secrete gonadotropic hormones that lead to an increase in LH and FSH levels [21]. LH stimulates the theca cells in the ovaries to produce androstenedione, whereas FSH promotes aromatase-dependent estradiol synthesis in follicular cells [22]. Estradiol plays an important role in the development of secondary sexual characteristics, such as thelarche, pubarche, and menarche in girls. Additionally, estradiol causes the pubertal growth spurt and accelerates bone maturation (Figure 2).

In our study, we evaluated the beneficial effect of hEIF extract against precocious puberty* in vivo* using SPF Sprague–Dawley rats. After 3 weeks of treatment, body growth indexes of the rats from all groups were determined. The rats were subsequently anesthetized to obtain blood samples for component analyses. The ameliorative effect of hEIF extract on precocious puberty was examined by comparing body weights, bone mineral densities, and gonadotropic hormone levels between the experimental and control groups, which are reflective of the changes in pubertal growth and endocrine function.

The body indexes and weights of organs measured after the 3-week treatment period in all groups are presented in Table 1. The body weight and body length except tail length increased slightly in the groups treated with hEIF extract, but there were no differences in the femur length between the control group and the test compound groups. The blood component analyses showed that treatment with hEIF extract caused a decrease in IGF-1 and IGFBP-3 levels, which stimulates proliferation and differentiation [23]. The ALP level and bone mineral density were not changed, implying that hEIF extract did not induce bone accretion (Table 2).

Activation of the HPG axis accelerates growth and bone maturation as a result of the increased synthesis and secretion of sex hormones, as well as growth hormones, such as serum IGF-1. As the rate of bone maturation increases, the epiphyses are fused early, and the final adult height is reduced to values lower than the target height. Thus, the goal of the treatment for precocious puberty is to match pubertal development of the patient's peers and to minimize the reduction in final adult height.

Several therapies, such as GnRH analogs, progesterone prescriptions, and herbal medicines, have been reported as effective treatments for precocious puberty. The standard treatment for precocious puberty is the use of gonadotropin-releasing hormone agonist (GnRHa). Synthesized GnRHa has a titer 20–150 times higher than that of GnRH present in the human body. Its mechanism of action is to suppress gonadotropin secretion through the desensitization of GnRH receptor expression in the pituitary gland [24]. For supporting treatment of precocious puberty, herbal medicines have been used to delay pubertal development and promote growth in patients with precocious puberty [25].

**Figure 1:**
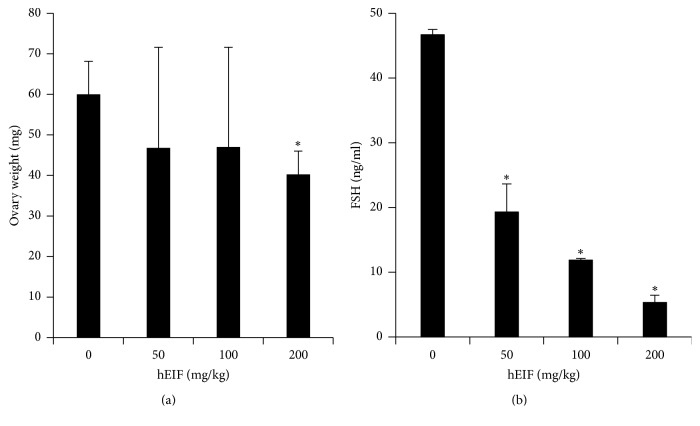
Effects of hEIF formulation on ovary weight and serum FSH concentrations of the control and test groups after 3 weeks of treatment. Sprague–Dawley rats with a mean body weight of 110 g were given hEIF extract for 3 weeks, and the effects on precocious puberty were identified. The prepared hEIF extract was administered orally at 50, 100, and 200 mg/kg BW to rats. ^*∗*^*p* < 0.05 compared with the control value. hEIF: herbal estrogen inhibition formulae. FSH: follicle stimulating hormone.

**Figure 2:**
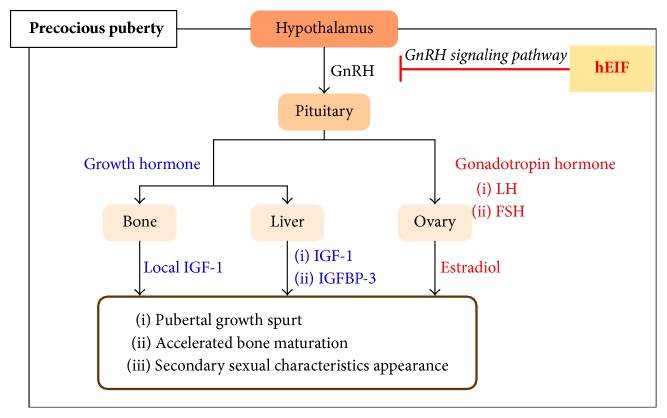
Hormonal mechanism scheme of precocious puberty. hEIF: herbal estrogen inhibition formulae.

Treatment with hEIF extract at 100 and 200 mg/kg decreased the weight of the ovary (Figure 1(a)). As shown in Table 2, the levels of LH and estradiol were not altered in the test groups compared to that in the control group. However, the level of FSH significantly decreased after treatment with hEIF extract (Figure 1(b)). The level of FSH in the blood decreased markedly after treatment with 200 mg/kg of hEIF extract to 5.33 ± 1.10 ng/mL in comparison with the level in the control group of 46.73 ± 0.80 ng/mL. The decreased FSH level in hEIF extract-treated groups showed that the oriental herbal formulation functioned as an FSH inhibitor for the prevention of precocious puberty in girls.

Next, network pharmacological analyses were performed to elucidate the complex relationships between the herbal compounds and their targets at the system level. Nine compounds from* Coicis Semen* and thirteen compounds from* A. capillaris* with good bioavailability (OB ≥ 30 and DL ≥ 0.18) were found. Targets of the compounds were predicted by using an in silico model, and the pathways involved were also investigated. On the basis of the KEGG pathway, with an adjusted *p* value of ≤0.05, identified annotation of the targets was 63 pathways for* Coicis Semen* and 153 pathways for* A. capillaris*. Among these pathways, we focused on the regulation of lipolysis and sex hormone-related pathways (estrogen signaling pathway, progesterone-mediated oocyte maturation, oxytocin signaling pathway, and GnRH signaling pathway). As shown in Tables 3 and 4, 9 and 22 genes were detected, which regulate the pathways of interest associated with* Coicis Semen* and* A. capillaris*, respectively.

**Figure 3:**
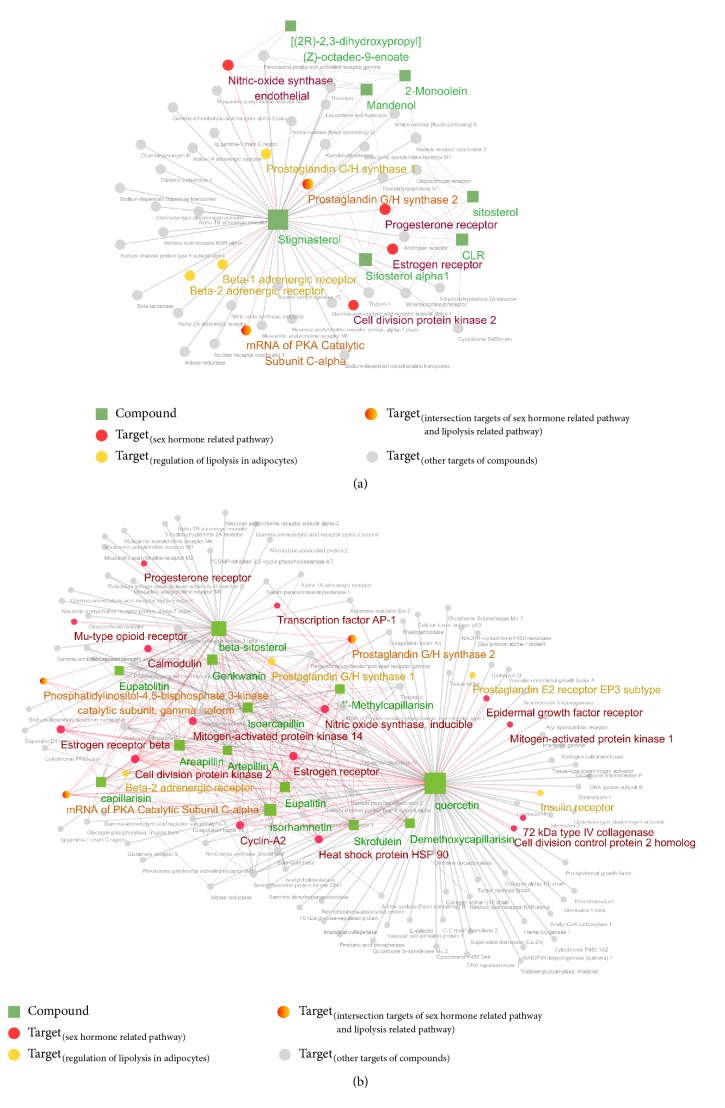
Compound–target networks of the component herbal formulation for precocious puberty. (a)* Coicis Semen*. (b)* A. capillaris*. Rectangles represent compounds (OB ≥ 30, DL ≥ 0.18), and circles represent the target genes. Nodes related to sex hormones or lipolysis regulation in the KEGG pathways are colored.

The compound–target networks were constructed and visualized using Cytoscape [26] to integrate the information about the compound–target interactions and pathways. The compound–target networks consisted of 54 nodes (9 compounds and 45 targets) and 87 edges for* Coicis Semen* and 126 nodes (13 compounds and 113 targets) and 395 edges for* A. capillaris*. The nodes and edges were colored to indicate the ones associated with the pathways of interest (Figure 3). Multiple targets related to lipolysis and sex hormone-related pathways of hEIF extract were discovered, which indicated the potential effects of* Coicis Semen* and* A. capillaris* for the treatment of precocious puberty.

As the results showed, treatment with hEIF extract at dose of 200 mg/kg significantly reduced the blood level of FSH in SPF Sprague–Dawley rats. More studies will be needed to clarify the role of hEIF extract in the mechanism of FSH release prevention. However, based on the network pharmacological analysis data, hEIF extract is expected to have impact on the GnRH signaling pathway. The binding between GnRH and its receptor on the cell surface of gonadotrope initiates the signaling cascades that lead to the synthesis of gonadotropins, FSH and LH. The production and secretion of FSH and LH are controlled by the transcription of the distinct *β*-subunits* Fshb* and* Lhb*, which are stimulated by GnRH such as mitogen-activated protein kinase cascades, calcium signaling, nuclear factor of activated T-cells transcription factor, and protein kinase A activity [27]. FSH not only promotes the estradiol synthesis in follicular cells, but also stimulates the subsequent growth of prenatal and antral follicles [28]. The decrease in FSH level in the animal group with hEIF extract might cause the difference of ovary weight with the control group through an effect on ovarian follicular development.

In conclusion, we have shown the preventive effects of hEIF extract on precocious puberty by measuring growth indexes and analyzing blood components. We also investigated the systems level mechanism of hEIF extract, which consists of multiple compounds with multiple targets by applying network pharmacological analysis, a novel in silico approach. On the basis of the* in vivo* experimental results, hEIF extract was identified as an effective therapeutic candidate for precocious puberty inhibitor. Its mechanism of action included reduction in the levels of gonadotropic hormones, such as FSH. Network pharmacological analysis revealed the potential bioactive compounds and their candidate targets, which turned out to be related to lipolysis and sex hormone-related pathways. Future studies will be conducted to confirm the molecular mechanism and phytochemical composition of hEIF extract that contributes to FSH inhibition.

## Figures and Tables

**Table 1:** Results of body index and weight of organs of the control and test groups after 3 weeks of treatment. ^*∗*^*p* < 0.05 compared with the control value.

Index	Control	hEIF extract		
		50 mg/kg	100 mg/kg	200 mg/kg
Body length except tail (cm)	17.87 ± 0.13	17.88 ± 0.29	18.08 ± 0.24	18.34 ± 0.14^*∗*^
Body length (cm)	36.53 ± 0.50	37.02 ± 0.44	37.02 ± 0.42	37.08 ± 0.32
Body weight (g)	172.83 ± 5.98	190.14 ± 4.23^*∗*^	189.46 ± 5.40^*∗*^	187.94 ± 4.56^*∗*^
Femur length (cm)	2.90 ± 0.05	2.91 ± 0.07	2.88 ± 0.02	2.92 ± 0.05
Femur weight (mg)	545.70 ± 23.16	540.80 ± 25.52	531.70 ± 29.46	544.80 ± 28.37
Kidney weight (mg)	1406.00 ± 87.08	1394.80 ± 57.29	1347.40 ± 90.85	1320.00 ± 84.10
Liver weight (mg)	7298.40 ± 828.43	7419.80 ± 25.52	7374.20 ± 884.96	6934.00 ± 306.75
Spleen weight (mg)	363.20 ± 49.11	422.80 ± 35.00	419.20 ± 40.07	385.80 ± 36.16

**Table 2:** Results of the blood component analysis of the control and test groups after 3 weeks of treatment. ^*∗*^*p* < 0.05 compared with the control value.

Index	Control	hEIF extract		
		50 mg/kg	100 mg/kg	200 mg/kg
ALP (ng/mL)	123.54 ± 4.81	126.19 ± 3.35	124.79 ± 4.28	119.89 ± 2.18
Bone mineral density (g/cm^2^)	0.090 ± 0.002	0.088 ± 0.003	0.088 ± 0.02	0.089 ± 0.003
Calcium (mg/dL)	2.33 ± 0.64	2.3 ± 0.59	2.38 ± 0.65	2.12 ± 0.50
Estradiol (pg/mL)	3.53 ± 0.21	3.54 ± 0.19	3.45 ± 0.35	3.12 ± 0.39
Glucose (mg/dL)	147.00 ± 24.24	167.80 ± 19.69	158.80 ± 19.40	175.80 ± 16.74
IGF-1 (ng/mL)	230.76 ± 12.12	197.91 ± 25.69	190.64 ± 20.17	185.82 ± 9.57
IGFBP-3 (ng/mL)	22.22 ± 1.05	19.65 ± 1.49	20.27 ± 0.45	21.1 ± 1.05
LH (mIU/mL)	292.33 ± 1.23	233.33 ± 1.63	293.4 ± 2.26	294.33 ± 2.13
Osteocalcin (pg/mL)	303.30 ± 4.79	306.90 ± 18.19	335.91 ± 14.46	326.83 ± 12.55
Total cholesterol (mg/dL)	62.50 ± 9.11	60.80 ± 7.26	56.60 ± 8.88	67.80 ± 10.83

**Table 3:** KEGG pathways related to sex hormones and lipolysis, significantly enriched (adjusted *p*-value of ≤0.05) by predicted targets of *Coicis Semen*.

Rank	KEGG pathway	Adjusted- *p* value	Genes (targets)
5/63	hsa04923: Regulation of lipolysis in adipocytes	3.86*E* − 06	Beta-1 adrenergic receptor; beta-2 adrenergic receptor; prostaglandin G/H synthase 2; mRNA of PKA catalytic subunit C-alpha; prostaglandin G/H synthase 1

39/63	hsa04914: Progesterone-mediated oocyte maturation	4.51*E* − 03	Cell division protein kinase 2; progesterone receptor; mRNA of PKA catalytic subunit C-alpha

40/63	hsa04915: Estrogen signaling pathway	4.52*E* − 03	Nitric oxide synthase; mRNA of PKA catalytic subunit C-alpha; estrogen receptor

48/63	hsa04921: Oxytocin signaling pathway	0.014	Nitric oxide synthase; mRNA of PKA catalytic subunit C-alpha; prostaglandin G/H synthase 2

**Table 4:** KEGG pathways related to sex hormones and lipolysis, significantly enriched (adjusted *p*-value of ≤0.05) by predicted targets of *Artemisia capillaris*.

Rank	KEGG pathway	Adjusted- *p* value	Genes (targets)
6/153	hsa04915: Estrogen signaling pathway	1.25*E* − 11	Heat shock protein HSP 90; transcription factor AP-1; nitric oxide synthase; 72 kDa type IV collagenase; mitogen-activated protein kinase 1; Mu-type opioid receptor; calmodulin; mRNA of PKA catalytic subunit C-alpha; estrogen receptor; epidermal growth factor receptor; estrogen receptor beta; phosphatidylinositol-4,5-bisphosphate 3-kinase catalytic subunit, gamma isoform

17/153	hsa04914: Progesterone-mediated oocyte maturation	5.57*E* − 08	Cyclin-A2; cell division control protein 2 homolog; cell division protein kinase 2; heat shock protein HSP 90; mitogen-activated protein kinase 1; mitogen-activated protein kinase 14; progesterone receptor; phosphatidylinositol-4,5-bisphosphate 3-kinase catalytic subunit, gamma isoform; mRNA of PKA catalytic subunit C-alpha

28/153	hsa04923: Regulation of lipolysis in adipocytes	2.11*E* − 07	Insulin receptor; prostaglandin E2 receptor EP3 subtype; beta-2 adrenergic receptor; prostaglandin G/H synthase 2; mRNA of PKA catalytic subunit C-alpha; phosphatidylinositol-4,5-bisphosphate 3-kinase catalytic subunit, gamma isoform; prostaglandin G/H synthase 1

57/153	hsa04912: GnRH signaling pathway	3.07*E* − 06	transcription factor AP-1; 72 kDa type IV collagenase; mitogen-activated protein kinase 1; mitogen-activated protein kinase 14; calmodulin mRNA of PKA catalytic subunit C-alpha; epidermal growth factor receptor

70/153	hsa04921: Oxytocin signaling pathway	9.66*E* − 06	Transcription factor AP-1; nitric oxide synthase; mitogen-activated protein kinase 1; calmodulin; mRNA of PKA catalytic subunit C-alpha; prostaglandin G/H synthase 2; epidermal growth factor receptor; phosphatidylinositol-4,5-bisphosphate 3-kinase catalytic subunit, gamma isoform
